# Sporadic Hemiplegic Migraine with ATP1A2 and Prothrombin Gene Mutations

**DOI:** 10.1155/2013/895057

**Published:** 2013-12-11

**Authors:** Jose Aceves, Diana Mungall, Batool F. Kirmani

**Affiliations:** ^1^Division of Pediatric Neurology, Department of Pediatrics, Scott & White Healthcare/Texas A&M, Health Science Center College of Medicine, 2401 S. 31st Street, Temple, TX 76508, USA; ^2^Texas A&M Health Science Center College of Medicine, Temple, TX 76508, USA; ^3^Epilepsy Center, Department of Neurology, Scott & White Neuroscience Institute/Texas A&M, Health Science Center College of Medicine, Temple, TX 76508, USA

## Abstract

*Background*. Hemiplegic migraine is a rare type of migraine that may present in children and adolescents. Both familial and sporadic hemiplegic migraines have similar prevalence and clinical characteristics. *Patient*. We report an adolescent with sporadic hemiplegic migraine who previously had a similar attack in the past and who was initially evaluated for a possible acute ischemic event. *Results*. Magnetic resonance angiography showed dilatation of the left middle cerebral artery that resolved in a follow-up study. She was also found to have a ATP1A2 (c.2273 G>C) mutation and a heterozygous prothrombin mutation. *Conclusions*. We suggest that patients with sporadic hemiplegic migraine be tested for both ATP1A2 mutations which in some cases may be pathogenic, and prothrombin mutations which increase the stroke risk for this patient population.

## 1. Introduction

Hemiplegic migraine is a rare subtype of migraine associated with transient hemiplegia [1] that occurs with equal prevalence in either sporadic or familial forms [1]. A motor aura is required for its diagnosis but it should be kept in mind that it is not the only type of aura occurring in the patient [2]. Sporadic hemiplegic migraine shares a similar clinical profile as familial hemiplegic migraine [3]. It is the only migraine disorder where pathogenetic mutations are known, since it is associated with mutations in the CANA1A, ATP1A2, and SCN1A genes [1]. Mutations in these genes have also been reported in some cases of sporadic hemiplegic migraine [4, 5]. Migraine has been reported to be a risk factor for ischemic stroke with possible mechanisms involving neuronal, vascular and coagulation abnormalities [6]. The case report presented in this paper describes a mutation in the ATP1A2 and prothrombin genes in a young adolescent with sporadic hemiplegic migraine. Mutations in the prothrombin gene may further increase the risk for stroke occurrence.

## 2. Case Report

A previously healthy 15-year-old girl was playing tennis when suddenly her speech became slurry and the right lower face and arm became numb. The family noticed a right facial droop and the patient realized that she could not grip the racquet with her right hand. The episode lasted 30 minutes. Subsequently, she developed a severe throbbing headache localized to the right frontal-temporal area accompanied by photophobia. In the local emergency room she underwent a computed tomography of the brain which was within normal limits. She was treated with aspirin and morphine and was transferred to our institution for further evaluation. Upon arrival, her neurological examination was normal. She reported a similar episode several years ago that was never evaluated. She also has a history of several episodes of migraine with aura and at 13 months of age she developed an acute cerebellar ataxia that resolved completely without treatment. Her family history is significant for maternal grandmother having lupus anticoagulant and her mother suffers from migraine with aura.

The patient was admitted to our institution where she underwent an extensive workup for stroke which included magnetic resonance imaging (MRI) of the brain and magnetic resonance angiography (MRA) of the circle of Willis. The MRI was normal and the MRA showed a dilatation of the left MCA. Thrombophilia workup revealed a heterozygous prothrombin G20210 mutation. She was also found to have a mutation (c.2273 G>C) of unknown significance in the ATP1A2 gene. She was discharged home on topiramate and a baby ASA. A repeat MRA as an outpatient 1 month later showed complete resolution of the dilatation of the left middle cerebral artery.

## 3. Discussion

This case report describes a young woman with sporadic hemiplegic migraine that was found to have a mutation of unknown significance (c.2273 G>C) in the ATP1A2 gene and a heterozygous prothrombin G20210A mutation. The patient has a history of migraine with aura and has experienced at least 2 episodes of a migraine with motor weakness which fulfills the diagnosis of sporadic hemiplegic migraine. There are no other family members affected with this condition. Sporadic hemiplegic migraine is part of the differential diagnosis in adolescents presenting with acute hemiparesis. It has similar clinical characteristics as familial hemiplegic migraine, where several mutations in the ATP1A2 gene have been found to be pathogenetic [7, 8]. Isolated mutations in this gene may participate in the pathogenesis of some cases of hemiplegic migraine [4]. This case had, in addition to a mutation in the ATP1A2 gene, a heterozygous prothrombin gene mutation, which so far has never been described in patients with sporadic hemiplegic migraine. It is well known that patients with migraine with aura have an increased risk of stroke [9]. Accordingly, the prothrombin gene mutation in this patient may further increase her risk for stroke.

This case illustrates the presence of a mutation in the ATP1A2 gene of unknown significance in a patient with sporadic hemiplegic migraine. At the present time we do not know if this mutation is pathogenic or not. It is likely that in the future more mutations in the ATP1A2 gene will be found in sporadic hemiplegic migraine supporting the notion that it is a genetic disorder. This case also illustrates the point that patients with sporadic hemiplegic migraine should have a thrombophilia workup to detect coagulation abnormalities that may further increase their risk for stroke.
